# Posterior arthroscopic subtalar arthrodesis without bone graft preserves hindfoot height and function

**DOI:** 10.1051/sicotj/2025054

**Published:** 2025-09-30

**Authors:** Nicolas Cellier, Lolita Micicoi, François Bauzou, Stanislas Marouby, Rémy Coulomb, Pascal Kouyoumdjian

**Affiliations:** 1 Department of Orthopedic Surgery, CHU Nîmes, Univ Montpellier Nîmes Cedex 9 France; 2 iULS-University Institute for Locomotion and Sports Pasteur 2 Hospital 06000 Nice France; 3 University Mechanical and Civil Engineering Laboratory UMR 5508 CNRS-UMCC 048 163 rue Auguste Broussonnet 34090 Montpellier France

**Keywords:** Subtalar arthrodesis, Arthroscopy, Hindfoot height, CT scan, Fusion

## Abstract

*Purpose*: This study aimed to assess hindfoot height (HFH) changes 12 months after posterior arthroscopic subtalar arthrodesis without bone grafting. We hypothesized that HFH reduction would be minimal and would not impact fusion or functional results. *Methods*: A retrospective study was conducted on 39 patients who underwent posterior arthroscopic subtalar arthrodesis. HFH was measured on CT scans preoperatively and at 12 months postoperatively. Inter- and intra-observer reliability of the measurement was also assessed as a secondary outcome. Clinical outcomes included pain (numeric analog scale, NAS) and AOFAS Ankle-Hindfoot scores. Subtalar fusion ratios were evaluated via CT. *Results*: Mean HFH loss was 0.85 ± 1.1 mm (range, 0–5 mm). The average fusion ratio was 72 ± 30%. Pain and AOFAS scores significantly improved (NAS: −4 ± 2, *p* < 0.0001; AOFAS: +31 ± 13, *p* < 0.0001). No correlation was found between HFH loss and fusion ratio or clinical outcomes. HFH loss > 1 mm was more frequent in women and smokers. HFH measurement on CT showed excellent inter- and intra-observer reliability (ICC intra: 0.989; inter: 0.976). *Conclusions*: Posterior arthroscopic subtalar arthrodesis without bone graft results in minimal hindfoot height loss, with no negative impact on subtalar fusion or functional outcomes. This technique reliably preserves hindfoot alignment and provides excellent clinical results. While the assessment of hindfoot height on CT demonstrated excellent inter- and intra-observer reliability, this was a secondary finding and supports the utility of CT-based measurements in the postoperative evaluation of subtalar arthrodesis.

## Introduction

Subtalar joint degeneration or subtalar osteoarthritis causes pain and instability [1]. It can result from various etiologies, including post-traumatic lesions, inflammatory disease, congenital malformations, or post-septic arthritis [2]. Subtalar fusion is considered after non-operative treatment failure, particularly articular infiltration, to limit pain associated with movement of the subtalar joint and improve functional status [3–5]. Several studies have demonstrated that arthroscopic subtalar arthrodesis provides comparable, if not superior, outcomes compared to open techniques, with fewer complications, faster consolidation, and quicker return to activity [4–6].

Open procedures are frequently combined with bone graft to enhance consolidation and preserve hindfoot height (HFH), which is required to allow efficient function of the midtarsal joint [7]. Posterior arthroscopic subtalar arthrodesis is considered on a well-aligned hindfoot, allowing the joint to be fixed in situ, without bone grafting. The subtalar joint is blocked with residual diastasis, primarily because the anterior subtalar joint is not abraded with this technique.

To date, very few studies have specifically examined hindfoot height (HFH) following posterior arthroscopic subtalar arthrodesis, and none have used a CT-based measurement method to quantify HFH variation postoperatively. This is important, as loss of HFH has been linked to altered biomechanics, anterior ankle impingement, and decreased functional outcomes in other types of arthrodesis [8–11].

It has been shown that CT scans were more suitable than X-rays for fusion analysis of hindfoot arthrodesis, as classical radiographs were frequently faulty [12]. CT-based assessment not only improves diagnostic precision but also enables accurate measurement of HFH, a parameter not routinely explored in the literature.

This study is relevant because the effect of posterior arthroscopic subtalar arthrodesis without bone grafting on hindfoot height, a factor influencing midfoot mechanics and long-term outcomes, is poorly documented. No previous work has combined CT-based hindfoot height measurement with both clinical and radiological results in this setting. By addressing this gap, this study may help guide decisions on graft use and fixation strategy.

We hypothesize that loss of HFH after posterior arthroscopic subtalar arthrodesis would be negligible or even non-existent. The study objectives were: 1. Is hindfoot height maintained 12 months after posterior arthroscopic subtalar arthrodesis without bone graft? 2. Is CT-based hindfoot height measurement reliable? 3. Does hindfoot height variation affect fusion ratio or functional outcomes?

## Material and methods

### Participants and study design

A retrospective, single-center study was conducted on 39 patients with subtalar osteoarthritis who underwent posterior arthroscopic subtalar arthrodesis by a single surgeon between September 2014 and October 2019. This analysis was done between January 2021 and April 2021 by an independent investigator. The exclusion criteria were follow-up < 12 months, arthrodesis revision, hindfoot malalignments > 5°, talofibular impingement (usually treated by open arthrodesis), history of ankle fusion or arthroplasty or double hindfoot arthrodesis, and improper operating protocol. A letter of non-opposition was sent to all the patients included, and this retrospective study was approved by our institutional review board. The average follow-up was 22 ± 12 months.

Forty-seven feet in 47 patients underwent surgery within the inclusion period, of whom eight were excluded from the analysis for ankle fusion (*n* = 3), total ankle arthroplasty (*n* = 1), single screw subtalar fusion (*n* = 1), or loss to follow-up (*n* = 3). Finally, 39 patients with an average age of 50 ± 15 years were included, with a total of 39 feet (Table 1). There was a male predominance (30M/9F), and most patients had experienced trauma, most commonly a calcaneus fracture (21/39, 54%).

**Table 1:** Demographic data of a 39-patient’ series of posterior arthroscopic subtalar arthrodesis. Data are presented as average ± standard deviation (range) or number (%).

	Patients *n* = 39
Age (years)	49.6 ± 14.9 (24–79)
Male / female ratio	30/9 (sex ratio = 3.3)
Follow-up (months)	21.7 ± 12.2 (12–57)
Etiology	
Trauma	31
• Calcaneus fracture	21 (54%)
• Talus fracture	7 (18%)
• Tibial pilon fracture	2 (5%)
• Hindfoot sprain	1 (2.5%)
Synostosis	2 (5%)
Chronic pain	2 (5%)
Rheumatoid arthritis	1 (2.5%)
Primary osteoarthritis	3 (8%)
Body Mass Index (kg /m^2^)	27.1 ± 5.3 (18–40)
Smoker (Yes/No)	14 (36%)/25 (64%)

### Surgical procedure

Posterior arthroscopy was performed in the prone position using a 4 mm, 30° arthroscope [10]. The surgical procedure for subtalar arthrodesis was consistent for all patients, as described and used in previous studies [11]. Anatomic landmarks, including the posterolateral and posteromedial portals, were marked. An arthroscope was inserted posterolaterally, and a shaver was introduced posteromedially, allowing for the gradual creation of a working chamber. Articular surface preparation was mainly done with multi-angled, spoon-shaped curettes. Chisels were used if needed for larger osteophytes. Arthrodesis was fixed using two divergent 6.5 mm compression screws (AutoFIX™ Stryker^®^), inserted through a short approach via the calcaneal tuberosity in the direction of the talus. The screws applied compressive forces across the joint, but residual diastasis remained. No bone grafts were performed. Weight bearing was prohibited for six weeks, with a plaster cast used for immobilization.

### Clinical assessment

Baseline demographic data, including age, sex, body mass index (BMI), tobacco consumption, and etiology, were collected. Clinical review was then carried out at 12 months’ follow-up. Functional scores were taken preoperatively and at 12 months’ follow-up using the American Orthopaedic Foot and Ankle Society (AOFAS) ankle-hindfoot score (pain, function, and alignment out of a total of 94 points, not considering items on hindfoot motion) [13]. Pain was also measured on a numerical analog scale (NAS) (0 = no pain, 10 = worst imaginable pain). Variation in NAS and AOFAS represented the difference between preoperative and postoperative scores.

### CT analysis

**Figure 1:**
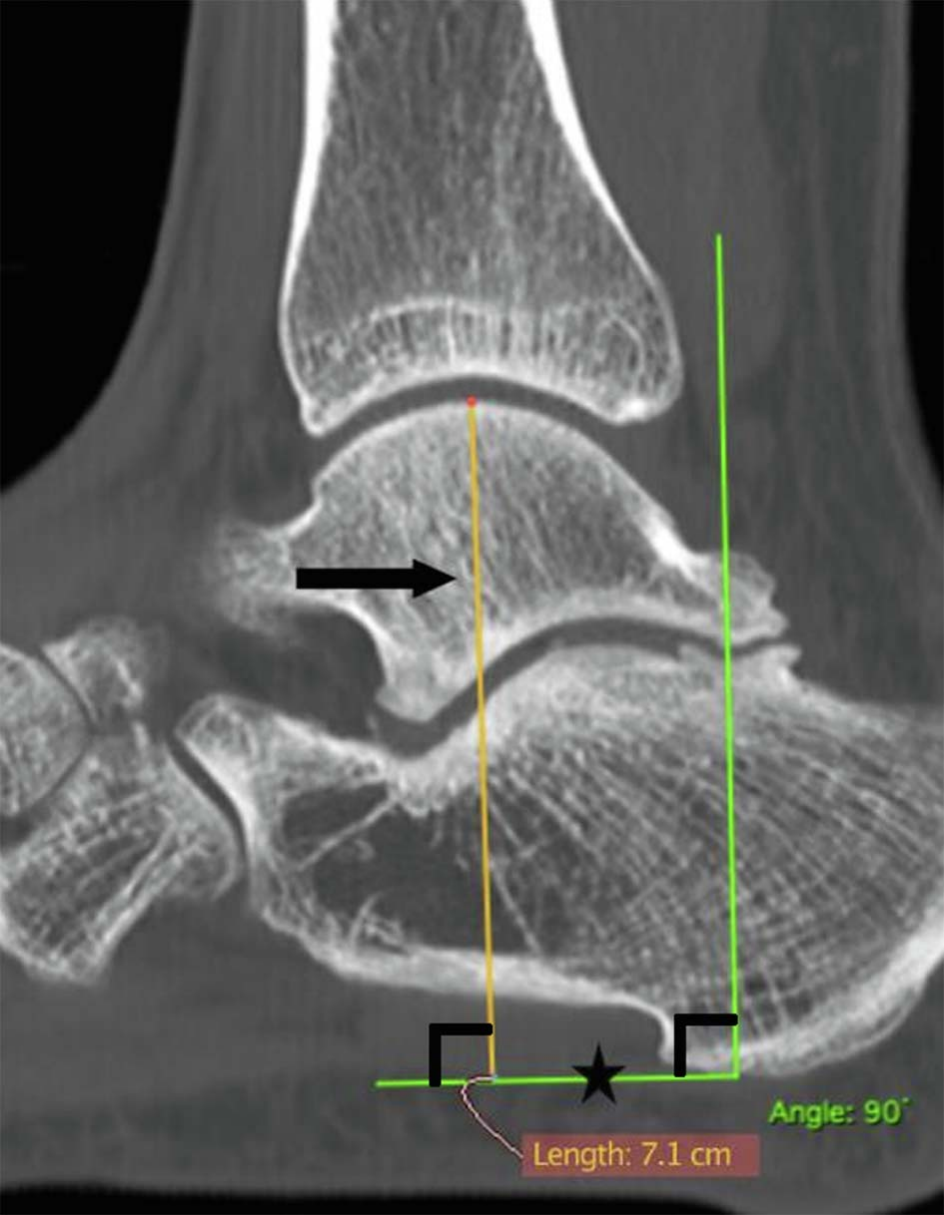
Preoperative hindfoot height (HFH) CT scan measurement. *Plantar fascia,* ⋆*; HFH, → .*

HFH CT scan measurement was performed on the median sagittal slice preoperatively and at 12 months. The median sagittal slice was precisely chosen using a multiplanar reconstruction system. HFH was defined as the length between the top of the talus and the most distal point of the calcaneus along a perpendicular line to the plantar fascia plane (Figure 1). Easy to identify on a CT scan, the plantar fascia is a constant reference point that represents the ground plane, thus justifying its use in our measurement method. HFH was measured independently by two reviewers, blinded to patient clinical data. One reviewer repeated the measurements 3 months later. HFH measurements were the mean of the three repeated measures. From these measurements, we calculated the length and percentage of HFH loss. Length of HFH loss was defined as LHFH-loss = HFH pre – HFH post. A LHFH-loss < 1 mm was assumed to be negligible. Indeed, according to a preliminary analysis on our data, the average difference between 2 intra-observer measurements was 0.63 mm, and the average variation between pre- and post-operative was 0.85 mm, thus explaining the 1 mm threshold retained earlier. At 12 months, CT scan fusion ratio was carried out on 2 mm sagittal slices. Fusion ratio was calculated for each cut, corresponding to the ratio of joint fusion length to the total length of the joint. These ratios were averaged by the number of slices as described in a previous study [14].

### Subgroup analysis

A subgroup analysis was performed to compare risk factors, fusion rates, and functional outcomes between an HFH loss length of > 1 mm and those without.

### Statistical analysis

Statistics were performed using a dedicated software (IBM SPSS Statistics, version 23.0.0.0). As the population did not follow a normal distribution, non-parametric tests were used. Quantitative variables were calculated with their averages, standard deviations, minimum and maximum values, and compared by Student’s test. Qualitative variables were described by their percentages and compared by Chi^2^ test. Pre- and postoperative quantitative variables were compared using a Student’s T-test for matched variables. The threshold significance was set at 0.05. Pearson’s intra-class correlation coefficients were calculated to define inter- and intra-observer variability for all measured parameters. They were accompanied by their 95% confidence intervals.

## Results

### Radiological results

The correlation of HFH CT scan measurement was strong with intra-observer (rho: 0.989 IC95% [0.98; 0.99]) and inter-observer (rho: 0.976 IC95% [0.96; 0.99]) (*p* < 0.0001). Intra- and inter-observer reliabilities were excellent, with respective *R*^2^ values of 0.979 and 0.952. Mean LHFH-loss was 0.85 mm ± 1.1 (0–5) (*p* < 0.0001). Nine cases (23%) showed both LHFH loss > 1 mm. Mean difference between two intra-observer HFH measurements was 0.63 mm ± 0.73 (0–2). Mean subtalar fusion ratio at 12 months’ follow-up was 72% ± 30 (0–98). There were three cases of non-union (7.7%), which successfully underwent open revision fusion with bone graft. Among them, there were two cases of talar enucleation. Two patients presented concomitant osteoarthritic evolution of the midfoot: one required complementary calcaneocuboid fusion. In both cases, it was midfoot post-traumatic osteoarthritis on the calcaneal side after calcaneus fracture without initial clinical repercussion, thus justifying isolated subtalar arthrodesis. Mean preoperative, postoperative HFH, and fusion ratio are shown in Table 2.

**Table 2:** Radiographic and clinical results after posterior arthroscopic subtalar arthrodesis. Comparison of pre- and post-operative radiographic (hindfoot height = HFH, subtalar joint fusion ratio) and clinical (Numerical Analog Scale = NAS for pain, AOFAS score) results.

*N* = 39	Preoperative	Postoperative	*p*-Value
Mean HFH (mm)	73.6 ± 6.2 (62.3–90)	72.8 ± 6.2 (61.7–90)	*p* < 0.0001
Mean fusion ratio (%)		72.1 ± 29.8 (0–98)	
Mean pain score (NAS)	6.6 ± 1.2 (4–9)	2.1 ± 1.9 (0–7)	*p* < 0.0001
Mean AOFAS score	50.9 ± 13.5 (13–69)	82.3 ± 10.2 (57–94)	*p* < 0.0001

### Clinical results

Clinical results were improved by 4 points ± 2 (−1 – 9) (*p* < 0.0001) for pain score and 31 points ± 13 (9–63) (*p* < 0.0001) for AOFAS score. Two complications (5.1%) were recorded: dysesthesia of the calcaneal branch of the tibial nerve, suggestive of lesions of the calcaneal branch of the tibial nerve, and seven cases (17.9%) of sural nerve dysesthesia. Among sural nerve dysesthesia cases, one required corticosteroid infiltration and one a surgical revision for neurolysis. We also reported three cases (7.7%) of complex regional pain syndrome. Two patients required hardware removal because of discomfort. All these complications had a positive outcome. Mean preoperative and postoperative pain (NAS) and AOFAS score are shown in Table 2.

### Subgroup analysis

There were no statistical differences between the extent of HFH loss (< 1 mm >) and age, BMI, fusion ratio, or improved NAS and AOFAS score (Table 3). In contrast, HFH loss was significantly greater in women and smokers.

**Table 3:** Subgroup analysis of delta hindfoot height loss > 1 mm after posterior arthroscopic subtalar arthrodesis. Significantly different variables are shown in bold. (HFH = Hindfoot height).

	HFH loss > 1 mm (*n* = 9)	HFH loss < 1 mm (*n* = 30)	*p*-Value
Age (years)	48.3	49.97	*p* = 0.797
Sex	5 M / 4 F	25 M / 4 F	***p* = 0.049**
BMI (kg/m^2^)	27.78	26.97	*p* = 0.707
Smokers	6 yes / 3 no	8 yes / 22 no	***p* = 0.028**
Fusion ratio (%)	64.89	74.3	*p* = 0.427
Pain NAS change	4.11	4.4	*p* = 0.765
AOFAS change	33.89	30.63	*p* = 0.567

## Discussion

Subtalar arthrodesis is a common surgical solution for advanced subtalar osteoarthritis when conservative treatment fails (Table 4).

**Table 4:** Summary of reported clinical and radiological outcomes of subtalar arthrodesis in the literature.

Investigator	Year	Methods	No. of patients	Union rate	Outcomes
Carranza-Bencano *et al*.	2013	MIS	76	92%	AOFAS: + 47.6 pointsSF-36: +14.5 points
Martín Oliva *et al.*	2017	Arthroscopic	19	94%	AOFAS: +37 pointsSF-12: +3.9
Vilá-Rico *et al*.	2017	Arthroscopic	65	95.4%	AOFAS: +51.5 points
Winson *et al.*	2005	Arthroscopic	116	92%	N/A
Smith *et al.*	2007	Open	23	99%	N/A

MIS: Minimal Incision Surgery; N/A: Not Available.

This study aimed to evaluate hindfoot height (HFH) changes after posterior arthroscopic subtalar arthrodesis using a reproducible CT-based measurement method.

We found that HFH loss was minimal (mean 0.85 ± 1.1 mm), that the method showed excellent inter- and intra-observer reliability, and that HFH variation was not associated with impaired functional outcomes or reduced fusion rates.

This study has several limitations, and they should be acknowledged early. First, the retrospective design and moderate sample size may introduce measurement bias and limit generalizability.

Second, no control group undergoing open arthrodesis was included for comparison, which it would have helped interpret the impact of the technique itself.

Third, the follow-up period was limited to 12 months, possibly missing later changes or complications.

Finally, the sex distribution was unbalanced (predominantly male), possibly underestimating the influence of osteoporosis on HFH loss.

Comparative analysis of posterior arthroscopic subtalar arthrodesis CT scans enabled us to propose an HFH measurement method, assess the rater concordance, and evaluate HFH variation pre- and postoperatively.

This supports our hypothesis concerning the very low compression in the fusion site. Subgroup analysis revealed a greater HFH loss among women and smokers, probably due to the increased frequency of osteopenia and/or osteoporosis in these populations [15, 16]. However, there were no statistical differences between the extent of HFH loss and functional results or fusion ratio.

In this study, we used an original measurement method based on CT scan, since this was performed systematically at 12 months postoperatively to assess fusion ratio [17]. Indeed, standard X-ray analysis does not appear to be reliable enough to assess fusion of the arthrodesis site [12]. Analysis of intra-class correlation highlighted excellent intra- and inter-observer reliabilities for our measurement method, justifying the application of this method for CT scan measurement of HFH.

Comparing radiographic results of isolated open subtalar arthrodesis with the healthy contralateral side, a significantly lower HFH was found on arthrodesis sides compared to healthy sides (7.16 ± 0.62 cm versus 7.74 ± 0.69 cm, *p* < 0.01). This result confirms the compression of the arthrodesis site in case of open surgery, unlike our posterior arthroscopic subtalar arthrodesis series. There was also no correlation between HFH and fusion ratio for subtalar arthroscopic fusion, confirming that posterior subtalar joint diastasis does not impede fusion. In contrast, sufficient HFH is required to ensure the strength of the gastrocnemius-soleus complex lever arm [18]. Correct HFH is also needed to limit anterior pain by anterior talar neck impingement on the distal tibia, which is due to horizontal talus position in case of serious loss of HFH [18, 19]. Few studies have focused on HFH variation. These data mainly come from subtalar distraction arthrodesis series with the use of bone graft or [20], more rarely, of porous tantalum implant [21]. The objective in these patients is to obtain a significant increase in HFH. These patients present significant hindfoot deformities requiring, in association with subtalar arthrodesis, hindfoot realignment, using bone graft or a specific implant. HFH variations in these cases cannot be compared with our series. Indeed, patients with hindfoot axis defect cannot be treated with an arthroscopic procedure and were not included in this series.

An alternative measurement is talocalcaneal divergence, mainly used to assess hindfoot deformities. Normalization of this divergence shows correction of these deformities [22]. However, we did not carry out this measurement, as our work focused on patients with correct hindfoot alignment. Weight-bearing cone beam CT scan (WBCT) in the foot and ankle allows more accurate measurement than weight-bearing radiograph series and conventional CT without weight-bearing [23, 24]. In the future, more widespread access to WBCT could help to reduce patient radiation dose, time spent on image acquisition, costs, and integrate the weight-bearing influence on HFH [25]. However, WBCT remains a rare device that was not available to us in this study.

This study focuses on HFH variation after posterior arthroscopic subtalar arthrodesis in a population of patients without hindfoot deformity. Although the HFH measurement technique remains open to criticism and requires validation in a prospective study, our analysis has shown a negligible loss in our series. This supports our hypothesis that compression is very low at the arthrodesis site, particularly due to the absence of anterior subtalar surface preparation in this technique. Our results are consistent with the existing literature [5] and confirm that posterior arthroscopic subtalar arthrodesis, fixed in situ with two screws without bone grafting, yields very satisfying clinical (NAS, AOFAS score) and radiological (CT fusion ratio and HFH) outcomes.

Hindfoot height plays a key role in the biomechanics of the ankle and hindfoot complex, particularly in ankle and subtalar arthrodesis. A sufficient HFH helps maintain the lever arm of the gastrocnemius-soleus complex, allowing effective push-off during gait and reducing compensatory overload on adjacent joints [26]. Loss of HFH has been associated with anterior impingement due to talar dorsiflexion, reduced ankle joint range of motion, and altered midfoot mechanics, which may contribute to degenerative changes over time [27]. Therefore, in both ankle and subtalar arthrodesis, it is essential to preserve variables such as hindfoot height, sagittal and coronal alignment, and talar tilt, as these factors collectively influence functional outcomes and long-term joint preservation [28]. The present findings, showing negligible HFH loss after posterior arthroscopic subtalar arthrodesis, suggest that this technique may help protect these critical biomechanical parameters compared with open procedures.

It has been shown in open subtalar arthrodesis series that HFH loss was related to ankle joint range of motion limitation [26, 29]. In a previous study of a bone block distraction arthrodesis series, a loss of ankle function in patients with dorsiflexed talus secondary to loss of HFH was already reported [30]. In this context, negligible HFH loss would limit talus dorsiflexion, reduce impact on joint kinetics of preserved midtarsal joints, and help to improve functional results after an arthroscopic procedure.

## Conclusion

Posterior arthroscopic subtalar arthrodesis without bone graft results in minimal hindfoot height loss, with no negative impact on subtalar fusion or functional outcomes. This technique reliably preserves hindfoot alignment and provides excellent clinical results. While the assessment of hindfoot height on CT demonstrated excellent inter- and intra-observer reliability, this was a secondary finding and supports the utility of CT-based measurements in the postoperative evaluation of subtalar arthrodesis.

## Data Availability

The data that support the findings of this study are available from the corresponding author upon reasonable request.
